# Supplementary material for: Inflammation and cytomegalovirus viremia during pregnancy drive sex-differentiated differences in mortality and immune development in HIV-exposed infants
Source: Nat Commun. 2024 Apr 17;15:2909. doi: 10.1038/s41467-023-44166-2 (PMC11024190; doi:10.1038/s41467-023-44166-2)
Supplement: Supplementary file 1 — Supplementary Information [file 41467_2023_44166_MOESM1_ESM.pdf]

Supplementary Table 1. Maternal, household, and infant baseline characteristics.

Baseline characteristics[1]	HIV-exposed	HIV-unexposed	*P*
Mothers [N]	726	3937	
Infants [N]	738	3989	
**Trial arm[2]**			0.17
SOC	166/738 (22%)	960/3989 (24%)	
IYCF	158/738 (21%)	963/3989 (24%)	
WASH	205/738 (28%)	996/3989 (25%)	
IYCF+WASH	209/738 (28%)	1070/3989 (27%)	
**Household characteristics**			
Size; median (IQR)	4 (3, 6)	5 (3, 6)	<0.001
Wealth quintile:[3]			<0.001
Lowest	191/712 (27%)	680/3659 (19%)	

Second	167/712 (23%)	706/3659 (19%)	
Middle	136/712 (19%)	743/3659 (20%)	
Fourth	106/712 (15%)	767/3659 (21%)	
Highest	112/712 (16%)	763/3659 (21%)	
**Maternal characteristics**			
Age, years; mean (SD)	29.2 (6.3)	25.6 (6.6)	<0.001
Height, cm; mean (SD)	160.2 (6.2)	160.1 (5.8)	0.84
MUAC, cm; mean (SD)	26.2 (2.9)	26.4 (3.1)	0.17
Completed schooling, years; mean (SD)	9.1 (2.1)	9.6 (1.8)	<0.001
Parity; median (IQR)	2 (1, 3)	2 (1, 3)	<0.001
Married	643/682 (94%)	3546/3717 (95%)	0.27
Employed	67/710 (9%)	311/3655 (9%)	0.38
Religion:			0.13
Apostolic	330/726 (45%)	1762/3937 (45%)	

Other Christian religions	288/726 (40%)	1685/3937 (43%)	
Other non-Christian religions	108/726 (15%)	490/3937 (12%)	
HIV disease severity and treatment:			
CD4 count in pregnancy, cells/uL; mean (SD)[4]	473.6 (221)	N/A	
CD4 count <200 cells/uL	46/613 (8%)	N/A	
Co-trimoxazole prophylaxis during pregnancy[5]	402/726 (55%)	N/A	
Antiretroviral therapy during pregnancy[6]	604/726 (83%)	N/A	
Tenofovir-based ART regimen	397/604 (66%)	N/A	
Zidovudine-based ART regimen	120/604 (20%)	N/A	
Other/unknown regimen[7]	87/604 (14%)	N/A	
**Infant characteristics**			
Female	367/733 (50%)	1962/3974 (49%)	0.73
Birth weight, kg; mean (SD)	2.99 (0.50)	3.08 (0.50)	<0.001
Birth weight <2500 g	84/651 (13%)	326/3574 (9%)	0.004

Institutional delivery	544/649 (84%)	3208/3604 (89%)	0.001
Vaginal delivery	610/659 (93%)	3411/3664 (93%)	0.64

[1] Baseline for mothers was 2 weeks after consent (~14 weeks gestation). Baseline for infants was at birth. Values are %, unless stated.

[2] SOC = standard of care; IYCF = infant and young child feeding; WASH = water and sanitation/hygiene.

[3] Wealth index constructed as described in (*1*).

[4] CD4 count at baseline visit, or at 32 gestational week visit if no baseline result.

[5] Documented exposure to co-trimoxazole during pregnancy.

[6] Documented exposure to antiretroviral therapy during pregnancy.

[7] Includes non-tenofovir- or zivoduine-based regimens, use of both tenofovir and zidovudine during pregnancy (including switching regimens), or undocumented antiretroviral therapy regimen.

SD: standard deviation; IQR: interquartile range; MUAC: mid-upper arm circumference.

Supplementary Table 2. Differences between median fluorescent intensities of CD40, CD86 and HLA-DR in monocyte subset between HEU and HIV-unexposed children at 1 month of age.

Monocyte marker, median fluorescent intensity	HIV-exposed uninfected	HIV-unexposed	Adjusted difference*	*P*
	Mean (95%CI)			
Classical monocyte CD40	6465.31 (6047.16, 6883.45)	6870.04 (6422.75, 7317.32)	-484.60 (-1023.74, 54.54)	0.078
Classical monocyte CD86	5205.29 (4767.28, 5643.29)	5430.24 (4736.53, 6123.95)	-281.57 (-1060.07, 496.93)	0.478
Classical monocyte HLA-DR	11 023.51 (9479.10, 12 567.92)	9917.15 (8612.45, 11 221.86)	1209.39 (-736.33, 3155.11)	0.223
Intermediate monocyte CD40	4142.83 (3871.74, 4414.92)	4155.45 (3852.22, 4458.69)	-42.82 (-439.02, 353.38)	0.832
Intermediate monocyte CD86	4483.77 (4146.56, 4820.98)	4413l98 (4009.08, 4818.88)	52.40 (-446.52, 551.33)	0.837
Intermediate monocyte HLA-DR	14 018.61 (12 214.84, 15 822.40)	12 351.40 (10 739.57, 13 963.24)	1654.3 (-594.35, 3903.00)	0.149
Non-classical monocyte CD40	2550.46 (2357.64, 2743.27)	4984.31 (86.68, 9881.95)	-2497.59 (-7395.49, 2400.31)	0.318
Non-classical monocyte CD86	2730.67 (2503.87, 4820.98)	2639.12 (2391.76, 2886.50)	102.60 (-219.75, 424.96)	0.533
Non-classical monocyte HLA-DR	9061.48 (7710.73, 10 412.23)	8163.29 (7115.91, 9210.66)	841.69 (-709.27, 2392.66)	0.287

Compared using linear regression models, fitted by generalised estimating equations with an exchangeable correlation to account for cluster. All statistical tests are two-sided, and presented P-values have not been adjusted for multiple comparisons.

*Adjusted for infant sex, exact age at time of blood collection, and randomised trial arm.

Supplementary Table 3. Association between second and third trimester HIV viral load in mothers living with HIV and T-cell and monocyte immunophenotyping of HIV children at 1 month of age.

	**Second trimester**				**Third trimester**			
	Unadjusted coefficient (95%CI)	*P*	Adjusted* coefficient (95%CI)	*P*	Unadjusted coefficient (95%CI)	*P*	Adjusted* coefficient (95%CI)	*P*
Naïve $CD8^+$ T-cells	0.02 (-0.10, 0.15)	0.746	0.00 (-0.13, 0.13)	0.998	-0.11 (-0.27, 0.05)	0.195	-0.12 (-0.29, 0.06)	0.187
Central memory $CD8^+$ T-cells	0.03 (-0.09, 0.15)	0.647	0.06 (-0.06, 0.18)	0.353	0.09 (-0.07, 0.26)	0.263	0.11 (-0.05, 0.28)	0.182
Effector memory $CD8^+$ T-cells	-0.08 (-0.22, 0.05)	0.223	-0.06 (-0.20, 0.07)	0.358	0.02 (-0.13, 0.17)	0.758	0.03 (-0.12, 0.19)	0.697
TEMRA $CD8^+$ T-cells	-0.05 (-0.16, 0.05)	0.342	-0.06 (-0.16, 0.05)	0.299	0.09 (-0.02, 0.20)	0.092	0.08 (-0.03, 0.19)	0.166
$CD28^+$ $CD57^-$ $CD8^+$ T-cells	0.02 (-0.08, 0.12)	0.719	-0.01 (-0.11, 0.09)	0.843	-0.11 (-0.24, 0.03)	0.117	-0.13 (-0.28, 0.10)	0.068
$CD28^-$ $CD57^+$ $CD8^+$ T-cells	0.00 (-0.10, 0.11)	0.950	0.02 (-0.09, 0.13)	0.744	0.16 (0.03, 0.29)	0.015	0.17 (0.04, 0.29)	0.010
$PD\text{-}1^+$ $CD8^+$ T-cells	0.03 (-0.07, 0.13)	0.552	0.04 (-0.06, 0.15)	0.430	0.10 (-0.02, 0.22)	0.117	0.11 (-0.02, 0.23)	0.096
$CD38^+$ $HLA\text{-}DR^+$ $CD8^+$ T-cells	0.00 (-0.12, 0.12)	0.964	0.09 (-0.04, 0.21)	0.170	0.16 (0.03, 0.29)	0.015	0.17 (0.04, 0.29)	0.010
$Ki67^+$ $CD8^+$ T-cells	0.05 (-0.07, 0.18)	0.419	0.05 (-0.07, 0.17)	0.431	0.10 (-0.02, 0.22)	0.117	0.11 (-0.02, 0.23)	0.096
Classical monocytes	0.03 (-0.03, 0.08)	0.311	0.02 (-0.04, 0.07)	0.537	0.10 (-0.07, 0.26)	0.244	0.12 (-0.05, 0.29)	0.167
Intermediate monocytes	0.01 (-0.05, 0.07)	0.674	0.02 (-0.04, 0.08)	0.527	0.04 (-0.15, 0.22)	0.698	0.06 (-0.13, 0.24)	0.557
Non-classical monocytes	-0.04 (-0.11, 0.03)	0.229	-0.03 (-0.10, 0.04)	0.353	0.06 (-0.02, 0.14)	0.138	0.05 (-0.03, 0.13)	0.260

Compared using fractional regression. All statistical tests are two-sided, and presented P-values have not been adjusted for multiple comparisons.

*Adjusted for gestational age at time of maternal blood sampling, exact infant age in days at time of infant blood sampling, infant sex, randomised trial arm.

Supplementary Table 4. Spearman correlations between maternal and infant $CD8^+$ T-cell and monocyte immunophenotypes.

	HIV-exposed uninfected		HIV-unexposed	
**Cellular phenotype**	**Spearman *rho***	***P***	**Spearman *rho***	***P***
$CD8^+$ naïve	0.26	0.028	-0.04	0.777
$CD8^+$ central memory	0.15	0.218	0.16	0.225
$CD8^+$ effector memory	0.21	0.088	0.00	0.978
$CD8^+$ TEMRA	0.16	0.190	0.05	0.682
$CD8^+$ $CD38^+$/HLA-DR^+	0.24	0.025	0.22	0.062
$CD8^+$ $Ki67^+$	0.29	0.008	-0.04	0.715
$CD8^+$ $CD28^+$/$CD57^-$	0.31	0.003	0.04	0.731
$CD8^+$ $CD28^-$/$CD57^+$	0.25	0.021	0.10	0.382
$CD8^+$ PD-1^+	0.07	0.526	0.20	0.080
Classical monocyte	-0.13	0.275	-0.12	0.341
Intermediate monocyte	-0.02	0.853	0.19	0.110

Non-classical monocyte	-0.03	0.815	0.00	1.00

Supplementary Table 5. Early CMV acquisition by HIV and CMV exposure

Exposure	Exposed	Unexposed	Odds ratio (95%CI)	*P*	Adjusted odds ratio (95%CI)	*P*
HIV	66/89 (74%)	48/100 (48%)	3.14 (1.63, 6.06)	0.001	3.00 (1.56, 5.76)*	0.001
Second trimester CMV	36/47 (77%)	72/132 (55%)	2.71 (1.22, 6.00)	0.014	1.41 (0.51, 3.87)**	0.510
Third trimester CMV	22/27 (81%)	57/104 (55)	3.61 (1.21, 10.76)	0.021	3.42 (1.13, 10.35)**	0.015

Compared using logistic regression. All statistical tests are two-sided, and presented P-values have not been adjusted for multiple comparisons.

*Adjusted for exact infant age at timing of saliva sample, infant sex, and randomised trial arm.

**Adjusted for maternal HIV, exact infant age at timing of saliva sample, infant sex, and randomised trial arm.

Supplementary Table 6. Differences in T-cell and monocyte phenotypes in HIV-unexposed children with and without early CMV acquisition.

	Mean percentage (95%CI)		Unadjusted coefficient (95%CI)	*P*	Adjusted* coefficient (95%CI)	*P*
	Early CMV acquisition (N=33)	CMV negative 3 months (N=39)				
Naïve $CD8^+$ T-cells	88.71 (83.94, 93.48)	93.67 (90.38, 96.95)	-0.32 (-0.66, 0.03)	0.072	-0.36 (-0.67, -0.05)	0.025
Central memory $CD8^+$ T-cells	7.04 (4.13, 9.94)	4.47 (2.77, 6.17)	0.23 (-0.04, 0.49)	0.096	0.22 (-0.01, 0.45)	0.059
Effector memory $CD8^+$ T-cells	1.31 (0.24, 2.39)	0.34 (-0.03, 0.71)	0.49 (0.03, 0.94)	0.038	0.48 (-0.10, 0.86)	0.014
TEMRA $CD8^+$ T-cells	2.95 (1.04, 4.86)	1.52 (-0.37, 3.42)	0.28 (-0.27, 0.82)	0.319	0.54 (0.11, 0.97)	0.015
$CD28^+$ $CD57^-$ $CD8^+$ T-cells	89.03 (84.53, 93.54)	91.33 (86.86, 95.81)	-0.13 (-0.49, 0.22)	0.462	-0.15 (-0.49, 0.20)	0.403
$CD28^-$ $CD57^+$ $CD8^+$ T-cells	3.32 (1.48, 5.16)	2.29 (0.47, 4.11)	0.16 (-0.24, 0.56)	0.429	0.15 (-0.23, 0.54)	0.426
PD-1^+ $CD8^+$ T-cells	3.69 (1.37, 6.01)	2.80 (0.85, 4.74)	0.12 (-0.28, 0.52)	0.544	0.13 (-0.24, 0.50)	0.502
$CD38^+$ HLA-DR^+ $CD8^+$ T-cells	7.40 (4.18, 10.61)	3.30 (1.31, 5.29)	0.39 (0.05, 0.73)	0.024	0.47 (0.17, 0.77)	0.002
$Ki67^+$ $CD8^+$ T-cells	10.95 (5.99, 15.90)	5.94 (3.26, 8.62)	0.33 (0.00, 0.66)	0.052	0.36 (0.05, 0.66)	0.021
Classical monocytes	61.86 (55.37, 68.35)	59.67 (54.38, 64.95)	0.06 (-0.15, 0.27)	0.591	0.07 (-0.13, 0.28)	0.484
Intermediate monocytes	13.12 (9.82, 16.42)	10.97 (8.79, 13.14)	0.11 (-0.08, 0.29)	0.254	0.11 (-0.06, 0.28)	0.217
Non-classical monocytes	25.02 (17.81, 32.23)	29.37 (24.24, 34.50)	-0.13 (-0.39, 0.13)	0.320	-0.15 (-0.40, 0.10)	0.239

Compared using fractional regression. All statistical tests are two-sided, and presented P-values have not been adjusted for multiple comparisons.

*Adjusted for gestational age exact infant age in days at time of infant blood sampling and saliva sampling, infant sex, and randomised trial arm.

Supplementary Table 7. CRP and IL-6 in HEU and HIV-unexposed children at 1 month of age according to infant sex.

	Sex	HIV-exposed uninfected, mean (SD)	HIV-unexposed, mean (SD)	Adjusted relative difference*	*P*
C-reactive protein (mg/L)	Female	2.79 (8.2)	3.32 (8.9)	0.65 (0.27, 1.53)	0.316
	Male	2.05 (7.0)	4.94 (16.5)	0.45 (0.19, 1.01)	0.053
Interleukin-6 (pg/mL)	Female	43.76 (79.1)	30.23 (52.8)	1.74 (0.51, 5.92)	0.369
	Male	24.40 (49.26)	36.50 (57.8)	0.83 (0.28, 2.49)	0.738

Compared using Tobit regression. All statistical tests are two-sided, and presented P-values have not been adjusted for multiple comparisons.

*Adjusted for exact age at time of blood collection and randomised trial arm.

Supplementary Table 8. Differences between proportions of monocyte and CD8+ T-cell subsets in HEU and HIV-unexposed children at 1 month of age according to infant sex.

		Female		Male	
	**Cell subset**	**Adjusted coefficient***	***P***	**Adjusted coefficient***	***P***
$CD8^{+}$ differentiation panel††	Naïve	-0.29 (-0.62, 0.04)	0.089	-0.45 (-0.79, -0.12)	0.009
	Central memory	0.19 (-0.09, 0.47)	0.186	0.31 (0.03, 0.58)	0.030
	Effector memory	0.37 (0.05, 0.74)	0.047	0.58 (0.21, 0.95)	0.002
	TEMRA	0.23 (-0.13, 0.59)	0.232	0.33 (-0.07, 0.74)	0.108
$CD8^{+}$ senescence/ exhaustion panel†††	CD28+/CD57-	-0.15 (-0.45, 0.15)	0.338	-0.46 (-0.78, -0.13)	0.006
	$CD28^{-}/CD57^{+}$	0.07 (-0.26, 0.40)	0.690	0.56 (0.18, 0.94)	0.004
	$PD\text{-}1^{+}$	0.09 -0.23, 0.42)	0.581	0.41 (0.05, 0.76)	0.024
$CD8^{+}$ activation/ proliferation panel†	CD38+/HLA-DR^{+}	0.36 (0.08, 0.64)	0.011	0.45 (0.12, 0.77)	0.007
	$Ki67^{+}$	0.22 (-0.09, 0.52)	0.166	0.43 (0.17, 0.69)	0.001
Monocyte panel**	Classical	-0.09 (-0.25, 0.07)	0.288	-0.16 (-0.31, -0.01)	0.040
	Intermediate	0.10 (-0.06, 0.26)	0.220	-0.02 (-0.15, 0.12)	0.815
	Non-classical	0.04 (-0.16, 0.23)	0.702	0.19 (0.00, 0.38)	0.050

Compared using fractional regression. All statistical tests are two-sided, and presented P-values have not been adjusted for multiple comparisons.

*Adjusted for exact age at time of blood collection. Classical monocytes: $CD3^-/CD19^-/CD20^-/CD56^-/HL\text{-}DR^+/CD14^{++}/CD16^-$; intermediate monocytes: $CD3^-/CD19^-/CD20^-/CD56^-/HLADR^+/CD14^{++}/CD16^+$; non-classical monocytes: $CD3^-/CD19^-/CD20^-/CD56^-/HLADR^+/CD14^+/CD16^{++}$. *Female HEU N=50, HIV-unexposed=47; male, HEU N=51, HIV-unexposed N=43. †Females HEU N=54, HIV-unexposed N=47; males, HEU N=54, HIV-unexposed N=44. ††Female HEU N=48, HIV-unexposed=40; male, HEU N=45, HIV-unexposed N=39. †††Female HEU N=54, HIV-unexposed=47; male, HEU N=54, HIV-unexposed N=44. TEMRA: terminally-differentiated effector memory.

Supplementary Table 9. Difference in T-cell and monocyte phenotypes in HIV-exposed uninfected children at 1 month of age with and without third trimester CMV exposure by infant sex.

	Male		Female	
	Adjusted* coefficient (95%CI)	*P*	Adjusted* coefficient (95%CI)	*P*
Naïve CD8$^+$ T-cells	-0.67 (-1.22, -0.11)	0.019	-0.51, -1.18, 0.17)	0.142
Central memory CD8$^+$ T-cells	0.31 (-0.08, 0.70)	0.123	0.18 (-0.33, 0.69)	0.487
Effector memory CD8$^+$ T-cells	0.58 (0.02, 1.14)	0.041	0.73 (0.05, 1.40)	0.034
TEMRA CD8$^+$ T-cells	0.69 (0.05, 1.33)	0.035	0.64 (0.20, 1.07)	0.004
CD28$^+$ CD57$^-$ CD8$^+$ T-cells	-0.66 (-1.11, -0.21)	0.004	-0.49 (-1.13, 0.14)	0.128
CD28$^-$ CD57$^+$ CD8$^+$ T-cells	0.95 (0.42, 1.47)	<0.001	0.71 (0.17, 1.26)	0.010
PD-1$^+$ CD8$^+$ T-cells	0.73 (0.22, 1.25)	0.005	0.59 (0.18, 1.00)	0.005
CD38$^+$ HLA-DR$^+$ CD8$^+$ T-cells	0.43 (0.04, 0.83)	0.031	0.19 (-0.42, 0.79)	0.546
Ki67$^+$ CD8$^+$ T-cells	0.05 (-0.43, 0.52)	0.847	0.24 (-0.44, 0.92)	0.492
Classical monocytes	0.13 (-0.12, 0.38)	0.310	-0.22 (-0.46, 0.03)	0.079
Intermediate monocytes	0.30 (0.01, 0.58)	0.041	0.25 (-0.13, 0.62)	0.198
Non-classical monocytes	-0.33 (-0.57, -0.09)	0.006	0.08 (-0.18, 0.35)	0.545

Compared using fractional regression. All statistical tests are two-sided, and presented P-values have not been adjusted for multiple comparisons.

*Adjusted for maternal HIV viral load, gestational age at time of maternal blood sampling, exact infant age in days at time of infant blood sampling, randomised trial arm.

Supplementary Table 10. Baseline characteristics of children meeting the selection criteria for the immunology sub-study.

Baseline characteristics[1]	HIV-exposed	HIV-unexposed	*P*
Mothers [N]	141	142	
Infants [N]	143	143	
**Trial arm[2]**			1.00
SOC	31/143 (22%)	31/143 (22%)	
IYCF	44/143 (31%)	44/143 (31%)	
WASH	35/143 (24%)	35/143 (24%)	
IYCF+WASH	33/143 (23%)	33/143 (23%)	
**Household characteristics**			
Size; median (IQR)	4 (3, 5)	4 (3, 6)	0.07
Wealth quintile[3]:			0.48
Lowest	34/140 (24%)	26/142 (18%)	
Second	31/140 (22%)	30/142 (21%)	
Middle	34/140 (24%)	36/142 (25%)	
Fourth	19/140 (14%)	31/142 (22%)	
Highest	22/140 (16%)	19/142 (14%)	
**Maternal characteristics**			
Age, years; mean (SD)	30.1 (6.5)	27.3 (6.7)	<0.001
Height, cm; mean (SD)	159.8 (5.5)	160.9 (5.6)	0.11
MUAC, cm; mean (SD)	26.2 (2.8)	27.2 (3.1)	0.008
Completed schooling, years; mean (SD)	9.0 (2.2)	9.6 (1.5)	0.03

Parity; median (IQR)	2 (1, 3)	2 (1, 3)	0.13
Married	123/134 (92%)	133/140 (95%)	0.36
Employed	12/138 (9%)	15/142 (11%)	0.59
Religion:			0.78
Apostolic	68/143 (48%)	65/143 (45%)	
Other Christian religions	61/143 (43%)	67/143 (47%)	
Other non-Christian religions	14/143 (10%)	11/143 (8%)	
HIV disease severity and treatment:			
CD4 count in pregnancy, cells/uL; mean (SD)[4]	471.4 (226)	N/A	
CD4 count <200 cells/uL	16/135 (12%)	N/A	
Co-trimoxazole prophylaxis during pregnancy[5]	100/141 (71%)	N/A	
Antiretroviral therapy during pregnancy[6]	136/141 (96%)	N/A	
Tenofovir disoproxil fumarate-based ART regimen	117/136 (86%)	N/A	
Zidovudine-based ART regimen	6/136 (4%)	N/A	
Other/unknown regimen[7]	13/136 (10%)	N/A	
**Infant characteristics**			
Female	74/143 (52%)	74/143 (52%)	1.00
Birth weight, kg; mean (SD)	3.02 (0.50)	3.17 (0.49)	0.005
Birth weight <2500 g	16/140 (11%)	12/138 (9%)	0.46
Institutional delivery	119/139 (86%)	126/141 (89%)	0.33
Vaginal delivery	131/141 (93%)	135/142 (95%)	0.45

[1] Baseline for mothers was 2 weeks after consent (~14 weeks gestation). Baseline for infants was at birth. Values are %, unless stated.

[2] SOC = standard of care; IYCF = infant and young child feeding; WASH = water and sanitation/hygiene.

[3] Wealth index constructed as described in (*1*).

[4] CD4 count at baseline visit, or at 32 gestational week visit if no baseline result.

[5] Documented exposure to co-trimoxazole during pregnancy.

[6] Documented exposure to antiretroviral therapy during pregnancy.

[7] Includes non-tenofovir- or zivoduine-based regimens, use of both tenofovir and zidovudine during pregnancy (including switching regimens), or undocumented antiretroviral therapy regimen.

SD: standard deviation; IQR: interquartile range; MUAC: mid-upper arm circumference.

Supplementary Table 11. Flow cytometry antibodies.

Target	Fluorochrome	Clone	Manufacturer	Catalogue number	Volume/tube
CD3	FITC	HIT3a	BD Biosciences	555339	20ul
CD4	APC-H7	SK1	BD Biosciences	641398	4ul
CD8	PECy7	SK3	BD Biosciences	641398	1ul
CD38	PE	HIT2	BD Biosciences	555460	20ul
Ki67	PerCP-Cy5.5	B56	BD Biosciences	561284	5ul
HLA-DR	APC	L243 (G46-6)	BD Biosciences	559866	20ul
CD27	PE	MT-271	BD Biosciences	555441	20ul
CD45RA	APC	HI100	BD Biosciences	550855	10ul
CD31	PerCP-Cy5.5	WM59	Biolegend	303132	5ul
CD57	PE	NK-1	BD Biosciences	560844	0.5ul
CD28	APC	CD28.2	BD Biosciences	559770	20ul
PD-1	PerCP-Cy5.5	EH12.1	BD Biosciences	561273	5ul
Anti-human lineage cocktail (CD3, CD19, CD20, CD56)	APC	UCHT1, HIB19, 2H7, 5.1H11	Biolegend	363601	5ul
CD66b	APC	G10F5	Biolegend	305118	2ul
CD16	APCCy7	3G8	Biolegend	302018	5ul
CD14	PE	HCD14	Biolegend	325606	2.5ul
HLA-DR	PE-Cy7	L243 (G46-6)	BD Biosciences	560651	5ul
CD86	FITC	BU63	Biolegend	374204	5ul
CD40	PerCP-Cy5.5	5C3	Biolegend	334316	2.5ul

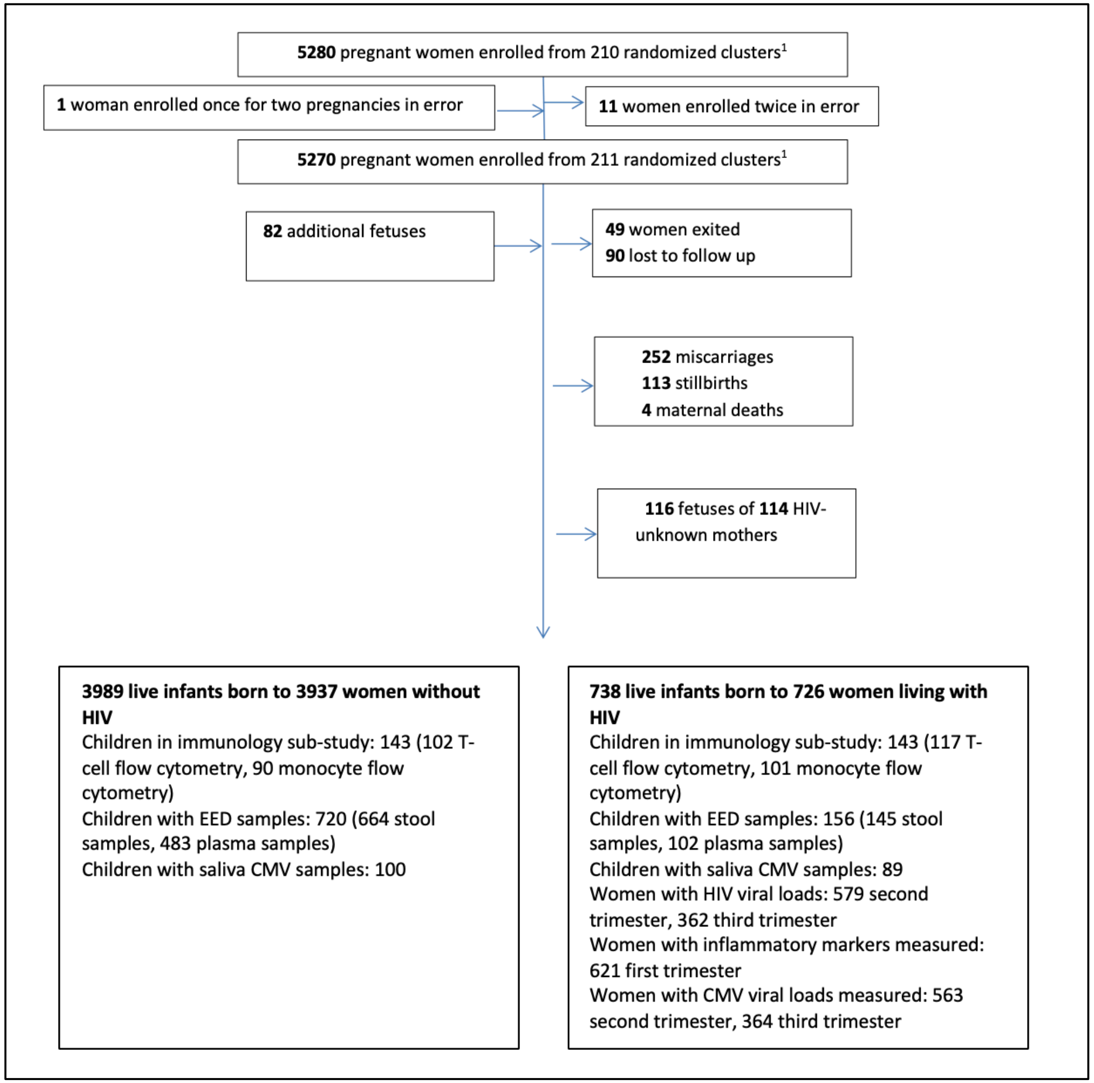

Supplementary Figure 1. Consort diagram showing the flow of participants through the SHINE trial.

[1]212 clusters were randomly assigned, 53 in each of the four trial groups. After randomisation, one cluster was excluded because it was in an urban area, one was excluded because the village health worker covering it mainly had clients outside the study area, and two more were

merged on the basis of subsequent data for village health worker coverage. Three new cluster designations were created because of anomalies in the original mapping. For two of these cluster, the trial group was clear; the third contained areas that were in two trial groups, and was assigned to the under-represented group, resulting in 53 clusters in each group. All these changes occurred before enrolment began. When enrolment was completed, however, no women were enrolled in one cluster in the SOC group and thus 211 clusters were available for analysis.

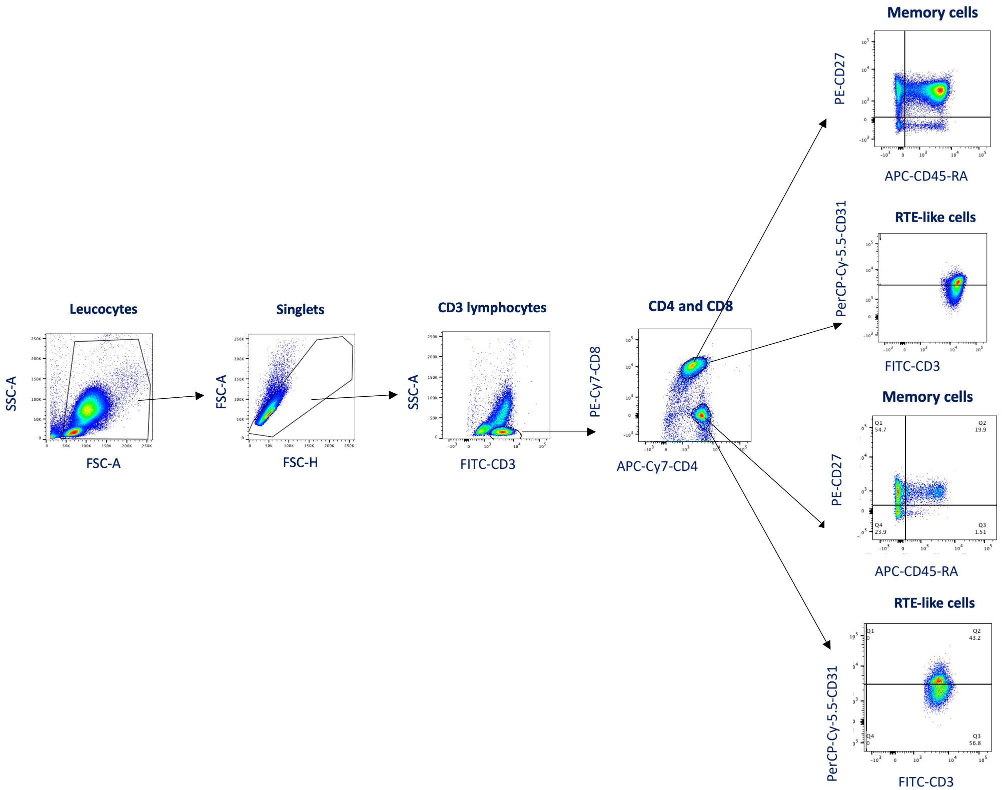

Supplementary Figure 2. Flow cytometry gating strategy for differentiation panel.

Sequential gating of cells to identify leucocytes, singlets, CD3+ T-cells, CD4+ T-cells and CD8+ T-cells, and immunophenotyping for naïve ($CD45RA^{+}/CD27^{+}$), central memory ($CD45RA^{-}/CD27^{+}$), effector memory ($CD45RA^{-}/CD27^{-}$), and terminally differentiated effector memory (TEMRA) ($CD45RA^{+}/CD27^{-}$) cells, and CD31+ recent thymic emigrant-like cells. RTE: recent thymic emigrant; SSC: side-scatter; FSC: forward-scatter.

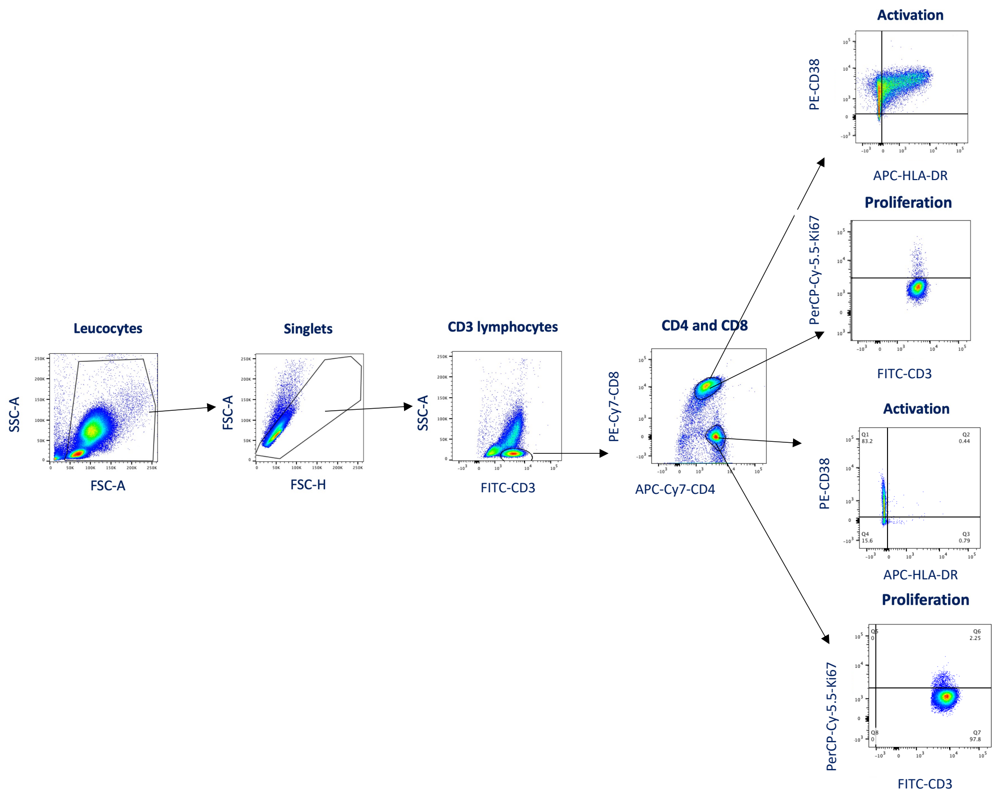

Supplementary Figure 3. Flow cytometry gating strategy for T-cell activation panel.

Sequential gating of cells to identify leucocytes, singlets, CD3+ T-cells, CD4+ T-cells and CD8+ T-cells, and immunophenotyping for activation ($CD38^+$/$HLA\text{-}DR^+$) and proliferating ($Ki67^+$) cells. SSC: side-scatter; FSC: forward-scatter.

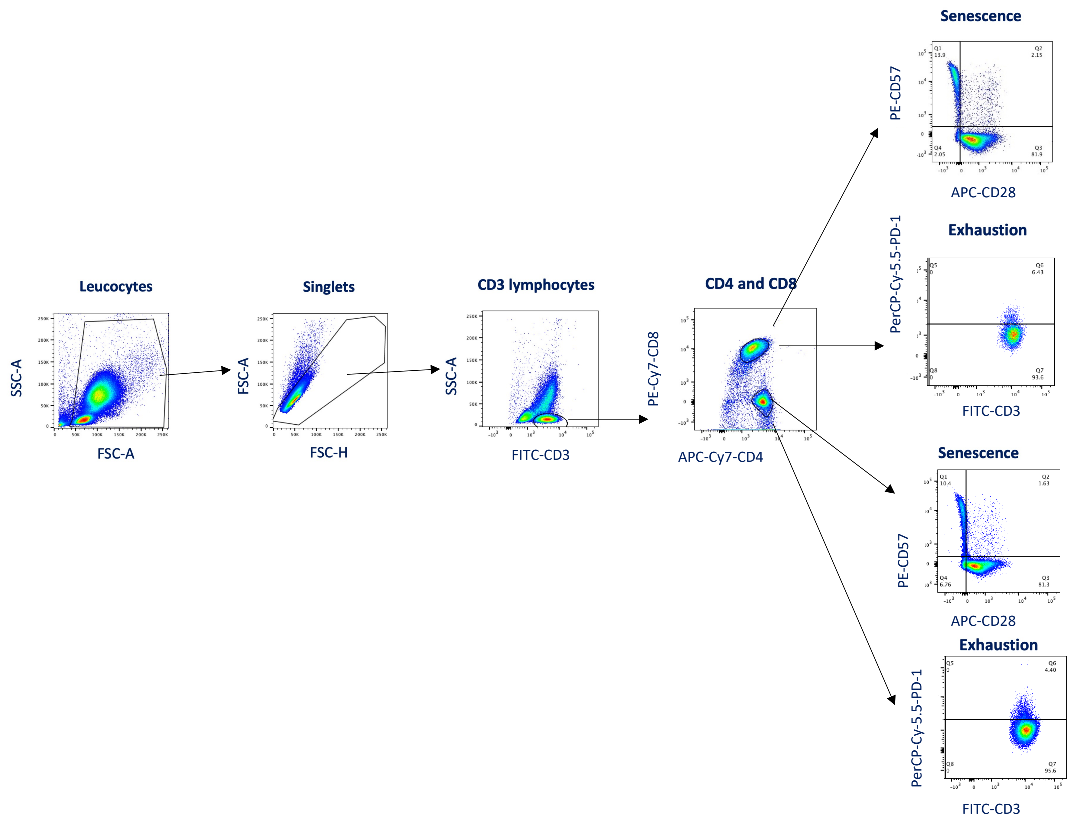

Supplementary Figure 4. Flow cytometry gating strategy for T-cell senescence/exhaustion panel.

Sequential gating of cells to identify leucocytes, singlets, CD3+ T-cells, CD4+ T-cells and CD8+ T-cells, and immunophenotyping for non-senescent ($CD28^{+}$/$CD57^{-}$), senescent ($CD28^{-}$/$CD57^{+}$), and exhausted ($PD\text{-}1^{+}$) cells. SSC: side-scatter; FSC: forward-scatter.

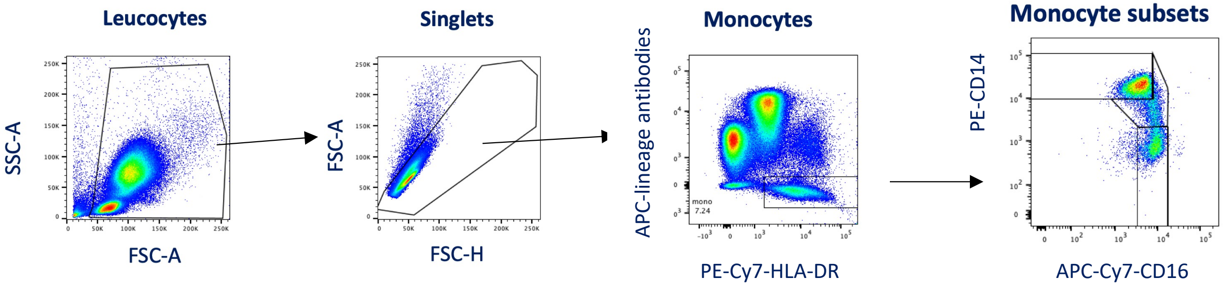

Supplementary Figure 5. Flow cytometry gating strategy for monocyte panel.

Sequential gating of cells to identify leucocytes, singlets and monocytes, and immunophenotyping for $CD14^{++}/CD16^{-}$ classical monocytes, $CD14^{++}/CD16^{+}$ intermediate monocytes and $CD14^{+}/CD16^{++}$ non-classical monocytes. SSC: side-scatter; FSC: forward-scatter.

Supplementary references

1. B. Chasekwa *et al.*, Measuring wealth in rural communities: Lessons from the Sanitation, Hygiene, Infant Nutrition Efficacy (SHINE) trial. *PLoS One* **13**, e0199393 (2018).
