# Supplementary material for: Inflammation and cytomegalovirus viremia during pregnancy drive sex-differentiated differences in mortality and immune development in HIV-exposed infants
Source: Nat Commun. 2024 Apr 17;15:2909. doi: 10.1038/s41467-023-44166-2 (PMC11024190; doi:10.1038/s41467-023-44166-2)
Supplement: Supplementary file 3 — Reporting Summary [file 41467_2023_44166_MOESM3_ESM.pdf]

nature portfolio

Corresponding author(s): Ceri Evans

Last updated by author(s): Aug 28, 2023

Reporting Summary

Nature Portfolio wishes to improve the reproducibility of the work that we publish. This form provides structure for consistency and transparency in reporting. For further information on Nature Portfolio policies, see our Editorial Policies and the Editorial Policy Checklist.

Statistics

For all statistical analyses, confirm that the following items are present in the figure legend, table legend, main text, or Methods section.

n/a	Confirmed	
☐	☒	The exact sample size (*n*) for each experimental group/condition, given as a discrete number and unit of measurement
☐	☒	A statement on whether measurements were taken from distinct samples or whether the same sample was measured repeatedly
☐	☒	The statistical test(s) used AND whether they are one- or two-sided<br>*Only common tests should be described solely by name; describe more complex techniques in the Methods section.*
☐	☒	A description of all covariates tested
☐	☒	A description of any assumptions or corrections, such as tests of normality and adjustment for multiple comparisons
☐	☒	A full description of the statistical parameters including central tendency (e.g. means) or other basic estimates (e.g. regression coefficient) AND variation (e.g. standard deviation) or associated estimates of uncertainty (e.g. confidence intervals)
☐	☒	For null hypothesis testing, the test statistic (e.g. *F*, *t*, *r*) with confidence intervals, effect sizes, degrees of freedom and *P* value noted<br>*Give P values as exact values whenever suitable.*
☒	☐	For Bayesian analysis, information on the choice of priors and Markov chain Monte Carlo settings
☒	☐	For hierarchical and complex designs, identification of the appropriate level for tests and full reporting of outcomes
☒	☐	Estimates of effect sizes (e.g. Cohen's *d*, Pearson's *r*), indicating how they were calculated

Our web collection on statistics for biologists contains articles on many of the points above.

Software and code

Policy information about availability of computer code

Data collection	No code used for data collection.
Data analysis	Stata v. 17.0 was used for all analyses. Covariables for multivariable Cox regression models were developed using direct acyclic graphs (DAG) using Dagitty software version 3.0 (www.dagitty.net). FACSuite was used for data collection. FlowJo version 10.7.1 was used for flow cytometry gating. Graphs were made using Stata v. 17.0 and GraphPad Prism v. 9.2.0.

For manuscripts utilizing custom algorithms or software that are central to the research but not yet described in published literature, software must be made available to editors and reviewers. We strongly encourage code deposition in a community repository (e.g. GitHub). See the Nature Portfolio guidelines for submitting code & software for further information.

Data

Policy information about availability of data

All manuscripts must include a data availability statement. This statement should provide the following information, where applicable:

- Accession codes, unique identifiers, or web links for publicly available datasets
- A description of any restrictions on data availability
- For clinical datasets or third party data, please ensure that the statement adheres to our policy

Questionnaire data were collected on written forms and doubly-entered onto an SQL database. The SHINE Trial data generated in this study have been deposited in the ClinEpiDB database under accession code https://clinepidb.org/ce/app/workspace/analyses/DS_0086998c2f/new/variables/EUPATH_0035127/

EUPATH_0044124. The SHINE Trial data are available under controlled access for tracking data usage, access can be obtained by submitting an access request then data access will be granted immediately upon request submission. The raw SHINE Trial data are protected and are not available due to data privacy laws. The processed SHINE Trial data are available at https://clinepidb.org.

Human research participants

Policy information about studies involving human research participants and Sex and Gender in Research.

Reporting on sex and gender	All data in this study are generated from pregnant/post-partum women and their children from 1-18 months of age. Findings have been reported by infant sex in a separate section in the Results and Discussion.
Population characteristics	Pregnant women 15-49 years living in Midlands province Zimbabwe; infants aged 0-18 months.
Recruitment	Recruitment procedures have been described in detail in the study protocol (https://osf.io/mgp3t/). Briefly, village health workers from the study area sensitized potential participants to the trial, provided pregnancy tests and eligible individuals were consented. However, these analyses were not the primary aims of the original trial. Laboratory analyses were reliant on sample collection, availability and storage. Bias could have been introduced from the mothers and infants with available samples, and technical factors relating to transportation and long-term cryopreservation may have affected laboratory assays. By design, infants who died prior to blood sampling at 1 month had no immunophenotyping data.
Ethics oversight	All SHINE mothers provided written informed consent. The Medical Research Council of Zimbabwe (MRCZ/A/1675), Johns Hopkins Bloomberg School of Public Health (JHU IRB # 4205) approved the study protocol. The SHINE trial is registered at ClinicalTrials.gov (NCT01824940).

Note that full information on the approval of the study protocol must also be provided in the manuscript.

Field-specific reporting

Please select the one below that is the best fit for your research. If you are not sure, read the appropriate sections before making your selection.

☒ Life sciences ☐ Behavioural & social sciences ☐ Ecological, evolutionary & environmental sciences

For a reference copy of the document with all sections, see nature.com/documents/nr-reporting-summary-flat.pdf

Life sciences study design

All studies must disclose on these points even when the disclosure is negative.

Sample size	Sample size calculations have previously been published for the larger SHINE trial from which this study is based (https://osf.io/mgp3t/). This sub-study uses a convenience sample set based on available samples. Before undertaking the study, we calculated that a sample of 300 infants would provide 90% power to detect differences of 0.38 standard deviations (SD) in each biomarker between infant groups, or 80% power to detect 0.33 SD. Sample selection is described in the Methods section.
Data exclusions	Mothers/infants were included in each experiment as sample availability allowed. We used a strict algorithm to determine HIV status, and mothers/infants were excluded when an accurate HIV status could not be determined.
Replication	Due to sample availability and cost limitations, experiments were carried out in singlicate.
Randomization	Cluster-randomization to the four trial arms was performed for the larger SHINE trial as previously detailed in the trial protocol (https://osf.io/mgp3t/).
Blinding	Due to the nature of the interventions (building pit latrines, hand-washing stations, etc.), the trial was unblinded. All laboratory work was undertaken blinded to exposure status (HIV, CMV, etc.). Flow cytometry gating was also blinded.

Reporting for specific materials, systems and methods

We require information from authors about some types of materials, experimental systems and methods used in many studies. Here, indicate whether each material, system or method listed is relevant to your study. If you are not sure if a list item applies to your research, read the appropriate section before selecting a response.

Materials & experimental systems

n/a	Involved in the study
☐	☒ Antibodies
☒	☐ Eukaryotic cell lines
☒	☐ Palaeontology and archaeology
☒	☐ Animals and other organisms
☐	☒ Clinical data
☒	☐ Dual use research of concern

Methods

n/a	Involved in the study
☒	☐ ChIP-seq
☐	☒ Flow cytometry
☒	☐ MRI-based neuroimaging

Antibodies

Antibodies used

Target Fluorochrome Clone Manufacturer Catalogue number Volume/tube
CD3 FITC HIT3a BD Biosciences 555339 20ul
CD4 APC-H7 SK1 BD Biosciences 641398 4ul
CD8 PECy7 SK3 BD Biosciences 641398 1ul
CD38 PE HIT2 BD Biosciences 555460 20ul
Ki67 PerCP-Cy5.5 B56 BD Biosciences 561284 5ul
HLA-DR APC L243 (G46-6) BD Biosciences 559866 20ul
CD27 PE MT-271 BD Biosciences 555441 20ul
CD45RA APC HI100 BD Biosciences 550855 10ul
CD31 PerCP-Cy5.5 WM59 Biolegend 303132 5ul
CD57 PE NK-1 BD Biosciences 560844 0.5ul
CD28 APC CD28.2 BD Biosciences 559770 20ul
PD-1 PerCP-Cy5.5 EH12.1 BD Biosciences 561273 5ul
Anti-human lineage cocktail (CD3, CD19, CD20, CD56) APC UCHT1, HIB19, 2H7, 5.1H11 Biolegend 363601 5ul
CD66b APC G10F5 Biolegend 305118 2ul
CD16 APCCy7 3G8 Biolegend 302018 5ul
CD14 PE HCD14 Biolegend 325606 2.5ul
HLA-DR PE-Cy7 L243 (G46-6) BD Biosciences 560651 5ul
CD86 FITC BU63 Biolegend 374204 5ul
CD40 PerCP-Cy5.5 5C3 Biolegend 334316 2.5ul

Validation

All antibodies are commercially available and have undergone quality checks/validation by the companies. We optimised each immunophenotyping panel prior to implementation in our study by comparing antibody labelling of fresh and cryopreserved buffy coat cell samples, and we titrated antibodies to increase the sensitivity for the affected marker.

Clinical data

Policy information about clinical studies

All manuscripts should comply with the ICMJE guidelines for publication of clinical research and a completed CONSORT checklist must be included with all submissions.

Clinical trial registration

NCT01824940

Study protocol

https://osf.io/w93hy

Data collection

Recruitment was conducted in Midlands province, Zimbabwe between Nov 22, 2012 and March 27, 2015. All follow-up data collection was completed by July 31, 2017. Briefly, village health workers from the study area sensitized potential participants to the trial, provided pregnancy tests and eligible individuals were consented. Data were collected in the home by study nurses. These study nurses visited mothers in early pregnancy (~2 weeks after consent) and at 32 gestational weeks, to assess maternal and household characteristics. At baseline, mothers had height, weight, and mid-upper arm circumference (MUAC) measured, and household wealth assessed. Infant birth date, weight, and delivery details were transcribed from health records. Home visits were scheduled at 1, 3, 6, 12, and 18 months postpartum, and anthropometry was measured and biological samples were collected. Mortality was assessed through home visits, village health worker reports, and telephone calls, with date of death recorded where available. Maternal ART use was documented at 3 timepoints (baseline, 32 gestational weeks, and 1 month postpartum) based on maternal report and review of handheld medical records.

Outcomes

Primary outcomes of the main SHINE trial (infant length-for-age Z score and haemoglobin concentrations at 18 months of age) have been previously outlined in the study protocol (https://osf.io/mgp3t/). Length-for-age Z-scores were assessed by comparing length and age of children against standardized World Health Organization growth standards. Haemoglobin concentrations were assessed using the Hemocue hemoglobinometer. Outcomes for this report were infant mortality, systemic inflammation, monocyte and T-cell immunophenotyping, markers of intestinal health, maternal CMV viraemia and infant CMV acquisition, which were secondary outcomes in the protocol. Infant mortality was assessed through study visits. Systemic inflammation and markers of intestinal health were assessed by ELISA. Monocyte and T-cell immunophenotyping were assessed using flow cytometry. Maternal CMV viraemia and infant CMV acquisition were assessed by polymerase chain reaction of maternal plasma and infant saliva, respectively.

Flow Cytometry

Plots

Confirm that:

☒ The axis labels state the marker and fluorochrome used (e.g. CD4-FITC).

☒ The axis scales are clearly visible. Include numbers along axes only for bottom left plot of group (a 'group' is an analysis of identical markers).

☒ All plots are contour plots with outliers or pseudocolor plots.

☒ A numerical value for number of cells or percentage (with statistics) is provided.

Methodology

Sample preparation	Blood was collected into EDTA tubes and centrifuged (5000 rpm for 5 minutes) to collect plasma and buffy coat cells. Buffy coats were mixed with 1.8mL of BD FACS lysing solution and incubated in the dark for 10 minutes. After a second centrifugation for 10 minutes at 400g, supernatant was discarded followed by resuspension, and 1mL cold freezing medium was added. The cryovial was stored at -80°C in a Mr. Frosty device, and the following day the cryovials were transferred to cryoboxes for ongoing storage at -80°C.<br><br>Cryopreserved buffy coat cells from each participant were thawed by swirling cryovials in water bath equilibrated to 37°C until an ice crystal remained. Liquid was washed twice with 1ml of phosphate-buffered saline (PBS)/10% fetal calf serum (FCS) and washings were transferred to clean FACS tubes. FACS tubes centrifuged at 900 revolutions per minute (rpm) for 5 minutes and supernatant discarded. Cells were resuspended in 500ul PBS/1% FCS, and split into 4 aliquots, and labelled with 4 panels of fluorescently-labelled antibodies to characterise T-cell activation, differentiation, senescence and exhaustion and monocyte phenotype. Cells were then with antibodies for 40mins in the fridge. For the Ki67 titration, the cells were washed in perm/wash buffer before washing in PBS/1% FCS. For all other titrations, 1ml of PBS/1% FCS was added per tube. FACS tubes were centrifuged at 900rpm for 5min and the supernatants discarded. Cells were then resuspended in 200ul PBS/1% FCS and run on the FACSVerse flow cytometer.
Instrument	BD FACSVerse.
Software	FACSuite for data collection; FlowJo version 10.7.1 for gating.
Cell population abundance	Samples were excluded if <3000 CD3+ T-cells were acquired or <1000 HLA-DR+ monocytes were acquired.
Gating strategy	Using a hierarchical gating strategy applied relative to fluorescence minus-one (FMO) controls for deciding positive or negative fluorophore, we first identified single leukocytes on the basis of side- and forward-scatter, and identified T-cells using CD3-FITC and then classified CD4+ (CD4/PE-Cy7) and CD8+ (CD8/APC-H7) T-cell differentiation (CD45RA/APC; CD27/PE; CD31/PerCP-Cy5.5), activation (CD38/PE; HLA-DR/APC) proliferation (intracellular Ki67-PerCP-Cy5.5, staining performed after membrane permeabilization), senescence (CD28/APC; CD57/PE) and exhaustion (PD-1/PerCP-Cy5.5). Using the same batches of cryopreserved cells, for monocytes, we first identified single leukocytes on the basis of side- and forward-scatter, then identified monocytes using anti-human lineage cocktail (CD3/CD19/CD20/CD56/APC) and HLA-DR/PE-Cy7, and then classified classical, intermediate and non-classical monocytes using CD14/PE and CD16/APC-Cy7.

☒ Tick this box to confirm that a figure exemplifying the gating strategy is provided in the Supplementary Information.
